# Histone 3 Methyltransferases Alter Melanoma Initiation and Progression Through Discrete Mechanisms

**DOI:** 10.3389/fcell.2022.814216

**Published:** 2022-02-10

**Authors:** Sara E. DiNapoli, Raúl Martinez-McFaline, Hao Shen, Ashley S. Doane, Alexendar R. Perez, Akanksha Verma, Amanda Simon, Isabel Nelson, Courtney A. Balgobin, Caitlin T. Bourque, Jun Yao, Renuka Raman, Wendy Béguelin, Jonathan H. Zippin, Olivier Elemento, Ari M. Melnick, Yariv Houvras

**Affiliations:** ^1^ Department of Surgery, Weill Cornell Medicine, New York, NY, United States; ^2^ Meyer Cancer Center, Weill Cornell Medicine, New York, NY, United States; ^3^ Division of Hematology/Oncology, Weill Cornell Medicine, New York, NY, United States; ^4^ Department of Medicine, Weill Cornell Medicine, New York, NY, United States; ^5^ Caryl and Israel Institute for Precision Medicine, Institute for Computational Biomedicine, Weill Cornell Medicine, New York, NY, United States; ^6^ Department of Anesthesia and Perioperative Care, UCSF, San Francisco, CA, United States; ^7^ Department of Dermatology, Weill Cornell Medicine, New York, NY, United States

**Keywords:** melanoma, histone methyl transferase (HMT), histone 3, epigenetics, zebrafish

## Abstract

Perturbations to the epigenome are known drivers of tumorigenesis. In melanoma, alterations in histone methyltransferases that catalyze methylation at histone 3 lysine 9 and histone 3 lysine 27—two sites of critical post-translational modification—have been reported. To study the function of these methyltransferases in melanoma, we engineered melanocytes to express histone 3 lysine-to-methionine mutations at lysine 9 and lysine 27, which are known to inhibit the activity of histone methyltransferases, in a zebrafish melanoma model. Using this system, we found that loss of histone 3 lysine 9 methylation dramatically suppressed melanoma formation and that inhibition of histone 3 lysine 9 methyltransferases in human melanoma cells increased innate immune response signatures. In contrast, loss of histone 3 lysine 27 methylation significantly accelerated melanoma formation. We identified *FOXD1* as a top target of PRC2 that is silenced in melanocytes and found that aberrant overexpression of *FOXD1* accelerated melanoma onset. Collectively, these data demonstrate how histone 3 lysine-to-methionine mutations can be used to uncover critical roles for methyltransferases.

## Introduction

Chromatin-modifying enzymes are subject to alterations in a diverse range of developmental disorders and cancers. Histone 3 lysine 9 (H3K9) methylation is a repressive histone modification that is catalyzed by the histone methyltransferases (HMTs) SETDB1, SUV39H1, G9A, and GLP. We have previously shown that overexpression of either SETDB1 or SUV39H1, two histone 3 lysine 9 (H3K9) methyltransferases, cooperate with oncogenic BRAF(V600E) to accelerate melanoma formation (Ceol et al., 2011). A subsequent study found that patients with melanoma, that were overexpressing another known H3K9 HMT, G9a, had poorer clinical outcomes (Miura et al., 2014). These studies underscore the importance of H3K9 methylation in tumorigenesis and malignancy.

Polycomb repressive complex 2 (PRC2) catalyzes methylation at histone 3 lysine 27 (H3K27) and is comprised of EZH2, EED, SUZ12 and RBBP4/7. PRC2 components are subject to gain and loss of function in a wide set of set of human cancers, including follicular and diffuse large B-cell lymphoma (Morin et al., 2010; McCabe et al., 2012), myelodysplastic syndromes (Nikoloski et al., 2010; Score et al., 2012), malignant peripheral nerve sheath tumors (De Raedt et al., 2014; Lee et al., 2014), and melanoma (Souroullas et al., 2016). These genetic studies demonstrate that the function of PRC2 as a tumor suppressor or oncogene is context dependent.

In addition to genetic alterations of chromatin-modifying enzymes, mutations in histones themselves have also been reported in pediatric neoplasms. The lysine-to-methionine (K-to-M) substitution at position 27 of histone H3.3 (H3.3K27M) has been identified in pediatric glioblastoma, diffuse intrinsic pontine glioma, and acute myeloid leukemia and has been shown to decrease levels of tri-methylation at H3K27 (H3K27me3) (Khuong-Quang et al., 2012; Schwartzentruber et al., 2012; Wu et al., 2012; Bender et al., 2013; Chan et al., 2013; Lewis et al., 2013; Venneti et al., 2013; Funato et al., 2014; Herz et al., 2014; Lehnertz et al., 2017). H3.3K27M has been shown to interact with the catalytic SET domain of EZH2 (Lewis et al., 2013; Justin et al., 2016) and leads to global loss of H3K27me3 when ectopically expressed at one percent of total histone levels (Lewis et al., 2013). Further assessment of K-to-M mutations at other residues demonstrated similar inhibitory activity; expression of histone H3.3 lysine 9K-to-M (H3.3K9M) reduced levels of H3K9 dimethylation (H3K9me2) and trimethylation (H3K9me3) (Herz et al., 2014) and H3.3K9M was shown to bind to the active site of G9a (Jayaram et al., 2016).

To study the function of these H3K9 and H3K27 methyltransferases in melanoma, we engineered melanocytes to express H3.3K9M and H3.3K27M in a zebrafish BRAF(V600E) melanoma model. Using this system, we found that loss of histone 3 lysine 9 methylation dramatically suppressed melanoma formation and that inhibition of histone 3 lysine 9 methyltransferases in human melanoma cells increased innate immune response signatures. In contrast, loss of histone 3 lysine 27 methylation significantly accelerated melanoma formation and led to deregulation of genes that are typically silenced by histone 3 lysine 27 trimethylation in melanocytes. Collectively, these data demonstrate how histone 3 lysine-to-methionine mutations can be used to uncover critical roles for methyltransferases.

## Materials and Methods

### Zebrafish Husbandry

Zebrafish were maintained according to established guidelines (Westerfield 2007). All studies were conducted under conditions approved by the Institutional Animal Care and Use Committee-(IACUC). Embryos were imaged using a Zeiss Discovery V8 stereomicroscope (Zeiss, Oberkochen, Germany).

### miniCoopR Assay

Full-length ORF for *H3F3B* was purchased from GE Healthcare Dharmacon. *EZH2*, *EZH2-Y641F* and *EZH2-Y641N* were a gift from Dr. Ari Melnick (Weill Cornell Medicine, New York, NY, USA). ORFs were PCR amplified and TA cloned into pCR8/GW and confirmed by Sanger sequencing (Genewiz, South Plainfield, NJ, USA). Site-directed mutagenesis was performed using QuikChange II XL Site-Directed Mutagenesis Kit (Agilent, Santa Clara, CA, USA). miniCoopR (MC) H3.3 and H3.3K9M constructs were created by MultiSite Gateway recombination (Invitrogen).

To generate transgenic animals, 25 pg of miniCoopR construct and 25 pg of Tol2 transposase mRNA were microinjected into Tg(mitfa:BRAF(V600E)); *tp53* ^zdf1/zdf1^; *mitfa* ^*w*2/w2^ or *tp53* ^zdf1/zdf1^; *mitfa* ^w2/w2^; zebrafish embryos at the one-cell stage. Embryos were scored for melanocyte rescue at 48 hpf and were raised to adulthood. Animals were observed weekly for melanoma from week 8 to 6 months.

### Tumor Processing

Tumor-bearing animals were sacrificed using 0.2% tricaine methanesulfonate and were imaged with a Canon EOS60D camera (Canon, Tokyo, Japan) prior to processing of tumor. Dissection of tumor was conducted using a scalpel and forceps, making every attempt to only include malignant tumor cells. Siliconized pipet tips (Bio Plas., Inc., San Rafael, CA, USA) were used for any pipetting for all assays to reduce the loss of material when processing.

### Histone Extraction From Zebrafish Tumors

Following dissection, tumors were transferred to tissueTUBE TT1 Extra Thick (Covaris, Wolburn, MA, USA), submerged in liquid nitrogen, pulverized using Covaris cryoPREP tissueTUBE Impactor CP02, and re-submerged in liquid nitrogen. Tumors were transferred to eppendorf tubes by resuspending in 1 ml cold PBS and centrifuging at 2000 RPM for 10 min at 4°C. Tumor pellets were resuspended in 250 μl TEB (0.5% Triton X-100 (MilliporeSigma) in PBS with Halt Protease Inhibitor Cocktail). Samples were lysed on ice for 20 min with intermittent shaking. Samples were then pelleted by centrifuging at 2000RPM for 10 min at 4°C, washed in 100 μl TEB, and centrifuging at 2000 RPM for 10 min at 4°C. To extract histones, samples were then resuspended in 60–200 μl 0.2 N HCl (MilliporeSigma) at 4°C on a nutator overnight. The next morning, samples were centrifuged at 2000 RPM for 10 min at 4°C and the supernatant was transferred to a new tube prior to storing at −80°C. Concentration of acid extracted histones was determined using Pierce Coomassie (Bradford) Protein Assay Kit (Thermo Fisher Scientific) and measured using Emax Plus Microplate Reader.

### RNA Extraction

To extract RNA from cells the RNeasy Mini kit (Qiagen) was used and the protocol was followed according to manufacturer’s instructions. Zebrafish tissue was processed as described in Anelli et al. (2017).

### RNA-seq

RNA-seq libraries were prepared according to the Illumina TruSeq RNA protocol. cDNA libraries were then run on the HiSeq platform to obtain 50 bp paired-end reads. Reads were then aligned to the zebrafish genome (GRCz10) using Star v2.3 (Dobin et al., 2013) using the Ensemble transcriptome (Howe et al., 2013). Differential gene expression was analyzed using DESeq2 (Love et al., 2014). For human gene orthology Ensembl was RNAseq data was used for GSEA and were also queried against the Hallmark in Cancer signatures from the MSigDB (http://www.broadinstitute.org/gsea/msigdb/index.jsp). Heatmaps were generated using Heatmapper and clustering method was average linkage and distance measurement method was Pearson (Babicki et al., 2016).

### Histopathology

Tissue was resected from the animal and fixed overnight in 4% PFA at 4°C. Tissues were then dehydrated in 70% ethanol and submitted to Histowiz, Inc (Brooklyn, NY, USA). The tissue was embedded in paraffin and 5 µm sections were placed onto slides and stained for H&E as well as PAS. IHC was performed for MPO per company protocol.

### Transposable Elements Analysis

The paired-end FASTQ files were aligned to the danRer10 genome using the bowtie2 aligner (Langmead et al., 2012). The BAM files had their reads counted using the HTSeq-count software. The GTF file for danRer10 transposable elements was taken from the UCSC genome browser under the “Variations and Repeats” group. The GTF file from ensembl was force converted compatible with HTSeq-count using a custom python script developed by the bioinformatician. The resulting count files were consolidated as a count table matrix and written to an output file using a custom python script developed by the bioinformatician. Count matrices were RPK filtered to reduce the hypothesis space and ameliorate the effect of excessive multiple hypothesis testing correction. Gene lengths and transposable element lengths used for RPK calculation were computed from the counting GTF files with a custom script developed by the bioinformatician. RPK threshold was set at 1 read per 1000 bases. Multiple hypothesis testing utilized the Benjamini-Hochberg correction. Statistical significance was assessed using the negative binomial test. Transposable elements were determined to be significant if their q-value was less than or equal to 0.05.

### Immunoblotting

0.25–5 μg of acid extracted histone lysate was prepared with Laemmli Sample Buffer (Bio-Rad Laboratories, Hercules, CA, USA) and β-mercaptoethanol (Bio-Rad Laboratories) and heated at 95°C for 5 min before transferring to ice to cool. Samples were then separated by SDS-PAGE on an 8–16% Mini-PROTEAN TGC Precast Protein Gel (Bio-Rad Laboratories) at 100 V for 60 min. Samples were transferred to 0.2 μM nitrocellulose membranes (Bio-Rad Laboratories) at 65 V for 45 min at 4°C and blocked in 5% milk in TBST (TBS with 0.1% Tween-20 (MilliporeSigma)). Blots were then incubated overnight in primary antibody diluted in milk with rotation at 4°C. The following antibodies were used for these studies: H2AK119Ub (8240S; Cell Signaling), H3K27me3 (07-449; Millipore), H3 (07-690; Upstate), H4 (ab7311; abcam), Flag (A8592; Sigma), H3K9me2 (ab1220; abcam), and H3K9me3 (8898; abcam). Blots were incubated in anti-mouse IgG (NA931; Millipore Sigma) or anti-rabbit (NA934; Millipore Sigma) for 1 h at room temperature. Immobilon Western Chemiluminescent HRP Substrate (MilliporeSigma) was used to visualize bands with a Konica SRX-101 X-ray film processor.

### Transfection and Cell Sorting

Synthetic genes of *H3F3B*-mScarlet (H3.3-mScarlet) and *H3F3B(K9M)-*mScarlet (H3.3K9M-mScarlet) were purchased from IDT provided in pUCIDT cloning vector. Genes were then PCR amplified and TA cloned into pCR8/GW and sequence confirmed. Constructs were then subcloned into pEF-DEST51 backbone using Gateway recombination (Invitrogen). Transfection of A375 cells were performed using Lipofectamine 3000 Reagent (ThermoFisher Scientific). Transfected cells were grown under selection (4 µg/ml blasticidin) 24 h post-transfection for 14 days. A375 cells were sorted using either a BD Influx or Sony MA900 sorter.

### Cell Culture and Growth Assays

B16-F10 and A375 cells were cultured in DMEM (Gibco) with 10% FBS (Denville Scientific). Cells were assessed for mycoplasma throughout the studies. For cell growth assays cells were seeded at 5000 cells per well in six-well plates using drug-free media and 24 h later media containing vehicle or compound was added. Cells were counted using a hemocytometer at days 1, 3 and 5 using trypan blue to exclude non-viable cells. For colony formation Cells were seeded at 500 cells per well in six-well plates using drug-free media and 24 h later media containing compound was added. Cells were cultured for 7–10 days and then fixed and stained with 0.2% crystal violet for colony visualization.

### Histone Extraction From Cell Lines

Cells were incubated in 200 µL TEB (0.5% Triton X-100 (MilliporeSigma) in PBS with Halt Protease Inhibitor Cocktail) for 10 min on ice. Lysates were then pelleted at 5000 RPM for 10 min at 4°C, washed with 100 µL TEB, and recentrifuged at 5000 RPM for 10 min at 4°C. Pellets were then resuspended in 250 µl TEB and lysed on ice for 20 min. Pellets were then resuspended in 80 µl of 0.2 N HCl and nutated overnight at 4°C. The next morning, samples were centrifuged and 5000 RPM for 10 min at 4°C and the supernatant was transferred to a new tube prior to storing at −80°C. Concentration of acid extracted histones was determined using Pierce Coomassie (Bradford) Protein Assay Kit (Thermo Fisher Scientific) and measured using Emax Plus Microplate Reader.

### Mouse Melanoma Model

All studies were conducted under conditions approved by the IACUC. Tyr::CreERT2;Braf^CA/wt^;Pten^fl/fl^ and Tyr:CreER;Braf^CA/wt^;Pten^fl/fl^;Ezh2^fl/fl^ mice were genotyped and treated with 4-hydroxytamoxifen (4-OHT) as previously described (Dankort et al., 2009). Briefly, mice were anesthetized with isoflurane (3%) and their backs were shaved to expose the treatment area. Approximately 10–15 µl of 4-OHT were painted on the exposed area for three consecutive days. Mice remained in the hazardous materials suite for 1 week and then transfered to the main animal facility.

## Results

### H3.3K9M Suppresses Melanoma Formation and Activates Innate Immune Response

**FIGURE 1:**
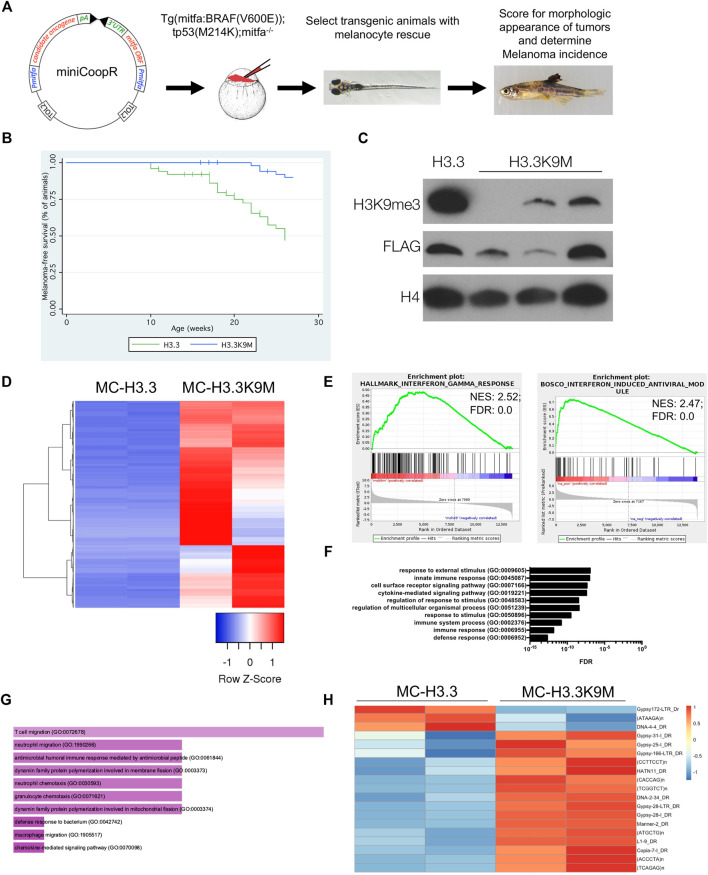
H3.3K9M suppresses melanoma formation. **(A)** The miniCoopR vector strategy overexpressing candidate genes in a melanocyte-specific fashion. Schematic adapted from Ceol et al. (2011). **(B)** Kaplan-Meier survival curves of Tg(mitfa:BRAF(V600E)); tp53^zdf1/zdf1^; mitfa^w2/w2^ zebrafish injected with indicated miniCoopR constructs (*p* value = 1.81e−06 calculated from log-rank test between miniCoopR H3.3 and H3.3K9M; 12 miniCoopR H3.3 and four H3.3K9M animals were censored from the study). **(C)** Western blot of acid-extracted histones from established tumors demonstrates a global loss of H3K9me3. **(D)** Top 1000 differentially expressed genes between miniCoopR H3.3 and H3.3K9M (sorted by *p*-value adjusted). **(E)** GSEA plots with enriched signatures in H3.3K9M tumors displaying enrichment for interferon responses. **(F)** Panther gene ontology analysis of the top 250 upregulated gene transcripts by *p*-value. **(G)** FishEnrichr analysis of the top 250 upregulated gene transcripts by *p*-value reveals neutrophil pathways among top 10 GO biological processes (Chen et al., 2013; Kuleshov et al., 2016). H, 16 of 19 transposable elements upregulated in H3.3K9M tumors.

To study the function of H3K9 methylation in melanoma, we used the miniCoopR system (Ceol et al., 2011) to overexpress wild-type H3.3 and H3.3K9M in Tg(mitfa:BRAF(V600E)); tp53^zdf1/zdf1^; mitfa^w2/w2^ zebrafish (Figure 1A). Given the known oncogenic function of H3K9 HMTs in melanoma, we hypothesized that H3.3K9M would suppress melanoma formation. By 27 weeks of observation, 35 of 53 (66%) miniCoopR H3.3 animals developed tumors. Of miniCoopR H3.3K9M animals, 45 of 54 (83%) were tumor-free at the 27 weeks of observation, indicating that H3.3K9M significantly suppressed melanoma formation (*p* = 1.81e−06, log-rank chi-squared test) (Figure 1B). H3.3K9M tumors had reduced levels of H3K9me2 and H3K9me3 (Figure 1C). Using cBioPortal (Cerami et al., 2012; Gao et al., 2013), we identified a single case of melanoma with a K9M mutation in histone 3, underscoring the importance of H3K9me3 in human melanoma.

To understand the underlying mechanism of H3.3K9M-mediated melanoma suppression, we performed RNA-seq analysis of miniCoopR tumors. Many of the top differentially expressed genes in H3.3K9M tumors were upregulated (Figure 1D), which is consistent with the known repressive effects of H3K9 methylation. Gene set enrichment analysis (GSEA) demonstrated enrichment of the innate immune response pathways, including interferon-induced antiviral response genes (Figure 1E). These results suggest that H3K9me3 is important in regulating immune response in melanoma and that loss of H3K9me3 leads to activation of the immune response.

To further uncover pathways altered in H3.3K9M melanomas, we performed gene ontology analysis of the top 250 significantly upregulated genes. Pathways involving responses to external stimuli and innate immune response were enriched in H3.3K9M tumors (Figure 1F). Using FishEnrichr analysis, we found neutrophil pathways were also enriched in H3.3K9M tumors (Figure 1G) Because H3K9 HMTs are known regulators of innate immunity through protection of transposable elements (Cuellar et al., 2017), we evaluated the expression of transposable elements in H3.3 and H3.3K9M tumors. There were 19 elements that were significantly differentially expressed, of which 16 (84%) were upregulated in the H3.3K9M tumors (Figure 1G). This indicates that H3.3K9M increases expression of a subset of transposable elements, thus activating the antiviral response and potentially playing another role in the innate immune response.

### Loss of H3K9 Methylation in Human Melanoma Cells Activates Immune Response

**FIGURE 2:**
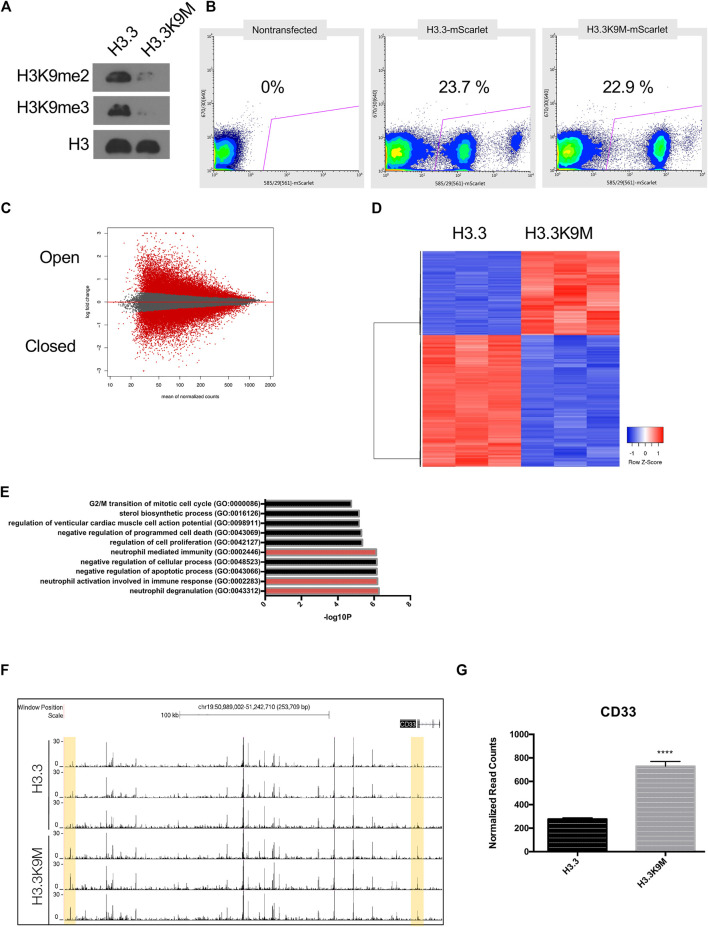
H3.3K9M induces interferon response. **(A)** Western blot of acid-extracted histones in A375 cells expressing H3.3 WT or H3.3K9M transgenes. **(B)** Percentage of mScarlet+ cells sorted from a heterogeneous H3.3-mScarlet and H3.3K9M-mScarlet cell population using non-transfected cell as mScarlet control. **(C)** MA plot of H3.3K9M vs H3.3 (Peaks that change at FDR <0.01 are colored red). **(D)** Top 1000 differentially expressed genes between H3.3-mScarlet and H3.3K9M-mScarlet (sorted by *p*-value adjusted). **(E)** Plot displaying Enriched signatures in H3.3K9M expressing A375 cells. Plot generated by GO Biological Process 2018 Enrichr (Mi et al., 2013). **(F)** ATAC-seq peaks that are differentially more accessible in H3.3K9M cells. **(G)** RNA seq normalized counts for CD33 in sorted cells (*n* = 4), (*p* < 0.0001).

H3K9 methylation is known to be important in maintenance of heterochromatin structure and transcriptional repression (Schultz et al., 2002), so we hypothesized that loss of H3K9 methylation would alter chromatin structure. To reduce H3K9 HMT activity, H3.3K9M-mScarlet was ectopically expressed in A375 melanoma cells. Global levels of H3K9me2/3 were reduced in H3.3K9M-mScarlet cells (Figure 2A). To isolate cells that express H3.3K9M, mScarlet-positive cells were sorted (Figure 2B). We evaluated chromatin accessibility in sorted cells by Assay for Transposase-Accessible Chromatin using sequencing (ATAC-seq). Sorted cells were processed using the OMNI-ATAC protocol and sequenced on the HiSeq platform to obtain 50 bp paired-end reads (Corces et al., 2017). H3.3K9M led to global changes in chromatin accessibility (Figure 2C), with 8,368 chromatin peaks more accessible and 8,165 less accessible in H3.3K9M cells (total peaks analyzed = 120,895). We also performed RNA-seq on sorted cells (Figure 2D) to identify transcriptional changes and identified enrichment of neutrophil degranulation pathways (Figure 2E). We sought to identify loci with significant changes in chromatin accessibility and transcription using our ATAC-seq and RNA-seq data, respectively. In H3.3K9M cells *CD33* was more accessible and expression was increased (*p* < 0.0001) (Figures 2F,G). CD33 is a transmembrane receptor that binds sialic acid that is part of the neutrophil degranulation pathway. These data suggest that *CD33* is a target of H3K9 methylation in melanoma cells.

### Inhibition of G9a/GLP Activates Immune Response Genes in Human Melanoma Cells

**FIGURE 3:**
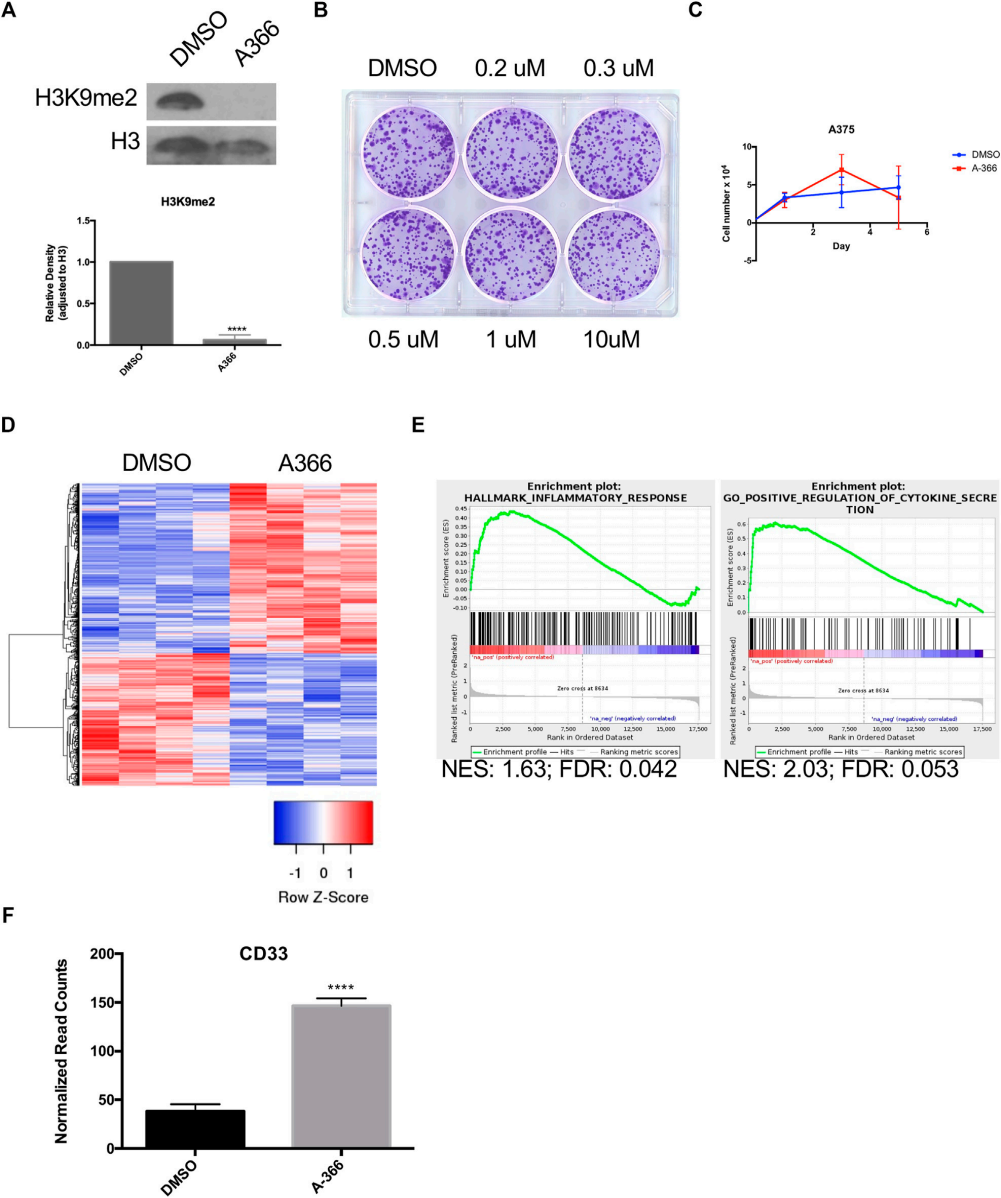
A-366 induces inflammatory response with no effect on cellular growth. **(A)** Western blot of acid-extracted histones from A375 cells incubated with either DMSO or 0.2 µM A-366 for 48 h. Densitometry analysis from four independent experiments. **(B)** A375 cells were treated with the indicated concentrations of A-366 for 10 days and assessed for colony formation. **(C)** Cell growth analysis of A375 cells treated with DMSO or A-366 (*n* = 3). **(D)** Top 100 differentially expressed genes between DMSO and A-366 treated cells (sorted by *p*-value adjusted). **(E)** GSEA plot showing enrichment in A-366 treated cells. **(F)** RNA seq normalized counts for CD33 in sorted cells (*n* = 4). (*p* < 0.0001).

Inhibitors of the H3K9 HMTs G9a/GLP have been shown to reduce H3K9me1/me2(Cao et al., 2019). A-366, a potent inhibitor with high selectivity and minimal toxicity, was chosen to treat A375 cells (Sweis et al., 2014; Pappano et al., 2015). A375 cells treated for 48 h with 200 nM of A-366 showed a significant reduction in H3K9me2 (Figure 3A). Treatment with A-366 did not alter colony formation or cell growth (Figures 3B,C). Transcriptional alterations post-treatment were evaluated using RNA-seq (Figure 3D). When performing GSEA there were gene sets that were significant for an immune response including cytokine secretion (Figure 3E). *CD33* was also significantly upregulated in cells treated with A-366 (*p* < 0.0001) (Figure 3F), further demonstrating that it is regulated by H3K9 methylation in melanoma.

### PRC2 Is Gained and Lost in Human Melanoma

**FIGURE 4:**
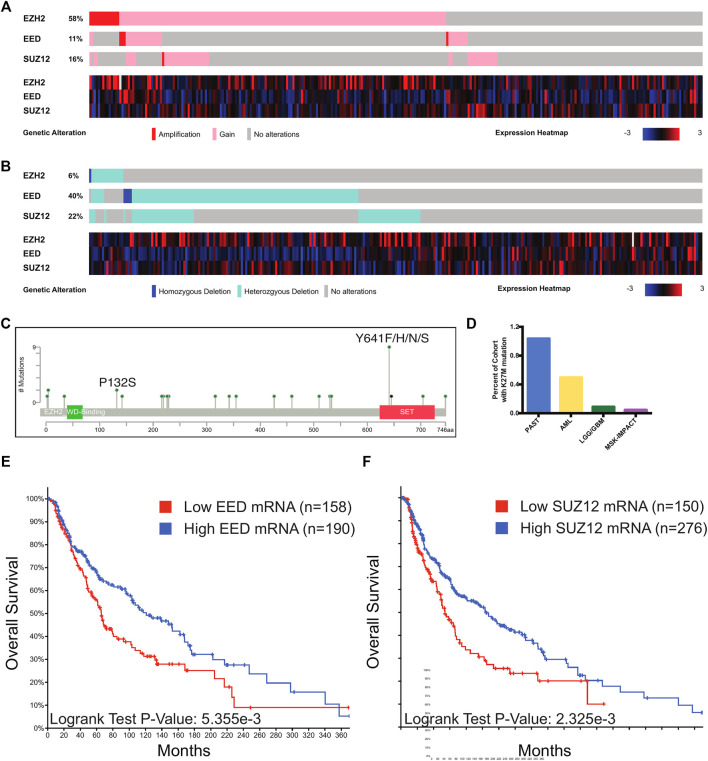
PRC2 alterations in melanoma. **(A**,**B)** Oncoprint output of TCGA melanoma data from cBioPortal. Top, chromosomal alterations in PRC2 components are shown. Below, heatmap with mRNA levels of PRC2 components. **(C)** Lollipop plot of mutations in EZH2. Recurrent mutations with known effects on catalytic activity are denoted. **(D)** Percentage of cases from TCGA cohorts with the K27M mutation in any H3 gene. PAST, pilocytic astrocytoma; AML, acute myeloid leukemia; LGG/GBM, low-grade glioma/glioblastoma. Of the five MSK-IMPACT cases, *n* = 1 diffuse intrinsic pontine glioma; *n* = 1 glioma; *n* = 1 high-grade glioma; *n* = 1 glioblastoma; *n* = 1 primary melanoma. **(E**,**F)** Stratification of survival of human melanoma patients based on EED **(E)** and SUZ12 **(F)** mRNA levels in TCGA melanoma cases with expression data. Cases with low EED expression have mRNA expression less than 0.6 standard deviations below the mean. Cases with low SUZ12 expression have mRNA expression less than 0.5 standard deviations below the mean. Plots generated with cBioPortal. *p*-value calculated from the Log-Rank Test.

In order to uncover the repertoire of PRC2 alterations in melanoma, we queried publicly available sequencing data from cohorts of primary and metastatic melanoma cases. We identified a proportion of melanoma cases which harbored chromosomal amplifications and gains of regions including *EZH2*, *SUZ12,* and *EED* (Figure 4A). Melanoma cases were found to contain homozygous and heterozygous deletions in *EZH2, SUZ12,* and *EED,* and cases with these alterations frequently had decreased mRNA levels of the deleted gene (Figure 4B). An analysis of *EZH2* point mutations identified the presence of gain-of-function Y641 substitutions and reduced activity P132S mutations in a small percentage of melanoma cases (Figure 4C). We also identified a single primary melanoma case which contained a H3K27 K-to-M substitution (Figure 4D), though at a lower frequency than observed in other tumor types such as astrocytoma. While this mutation has previously been reported in pediatric glioblastomas and acute myeloid leukemia, this is the first report of a H3K27M mutation in melanoma. Human melanoma patients with decreased *EED* or *SUZ12* mRNA have decreased overall survival (Figures 4E,F). These genetic data demonstrate that melanoma cases harbor both gain and loss of function alterations in PRC2.

### H3.3K27M Accelerates Melanoma Formation

**FIGURE 5:**
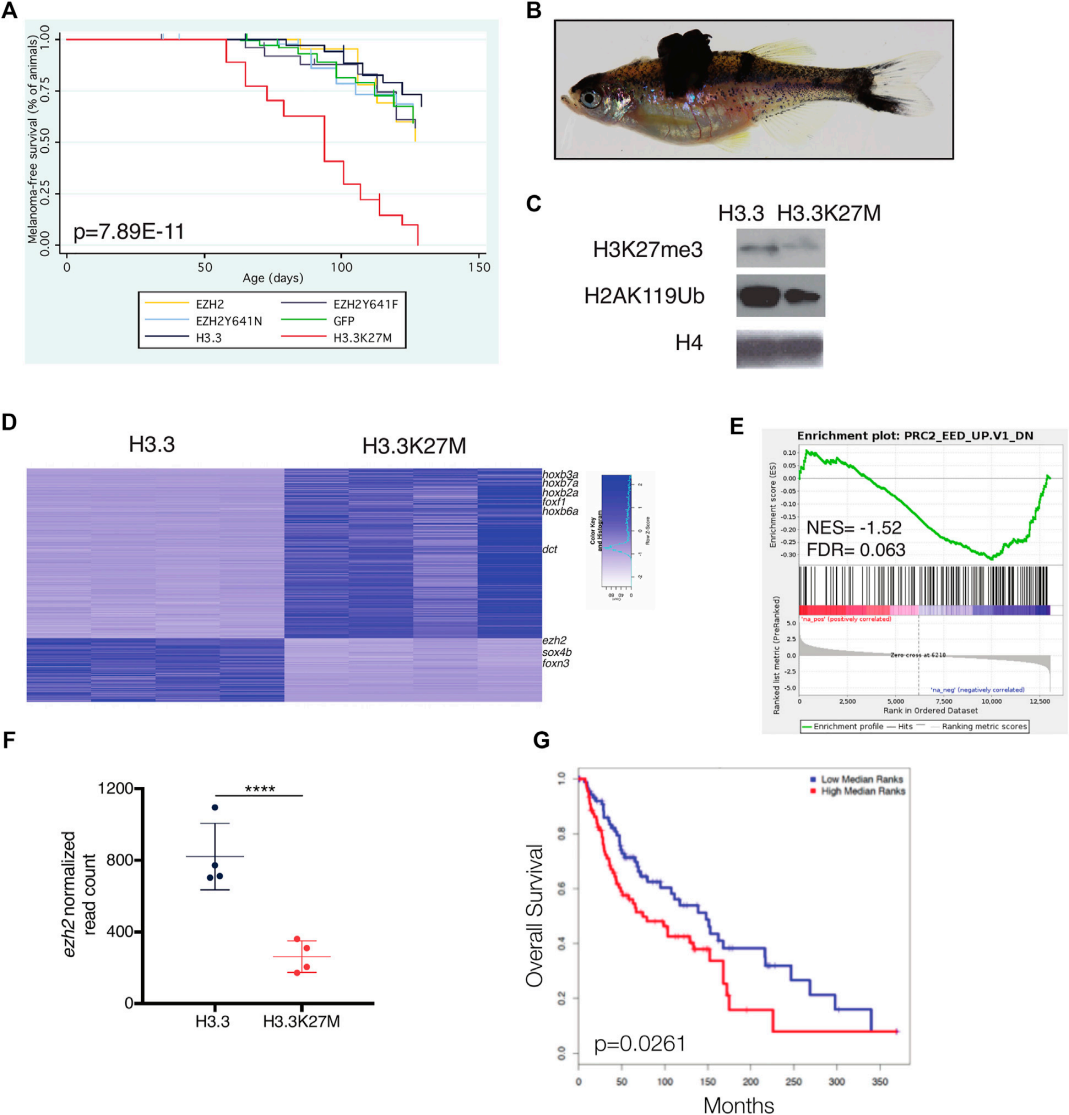
H3.3K27M accelerates melanoma formation. **(A)** Kaplan-Meier survival curves of Tg(mitfa:BRAF(V600E)); tp53^zdf1/zdf1^; mitfa^w2/w2^ zebrafish injected with indicated miniCoopR constructs. *p* value shown calculated from log-rank test between miniCoopR H3.3 and H3.3K27M. **(B)** Representative tumor-bearing miniCoopR H3.3K27M animal. **(C)** Western blot of indicated histone marks from miniCoopR H3.3 and H3.3K27M tumors. **(D)** Top differentially expressed genes between miniCoopR H3.3 and H3.3K27M. Genes with differential expression p < 1E05 are shown. **(E)** Gene set enrichment analysis (GSEA) of miniCoopR H3.3 and H3.3K27M tumors. NES, normalized enrichment score; FDR, false discovery rate; F, normalized read count of *ezh2* from miniCoopR H3.3 and H3.3K27M tumors. *p* = 6.37E-06, log_2_ fold change = −1.52. **(G)** overall survival of human melanoma patients (*N* = 180) stratified into low (blue) or high (red) expression of 50 gene signature developed from miniCoopR H3.3K27M tumors.

To elucidate the role of PRC2 in melanoma, we overexpressed *EZH2*, *EZH2*-Y641F, *EZH2*-Y641N, H3.3, and H3.3K27M using miniCoopR. We found that H3.3K27M accelerates melanoma onset as compared to wild-type H3.3 (Figures 5A,B, *p* = 2.42E-11 log-rank chi squared test). H3.3K27M failed to initiate melanoma formation in tp53^zdf1/zdf1^; mitfa^w2/w2^ animals (Supplementary Figure S1). Our results demonstrate that this model of reduced PRC2 activity accelerates melanoma onset.

### Gene Expression Changes in H3.3K27M Zebrafish Melanomas Predict Survival of Human Patients

Consistent with reduced PRC2 activity, H3.3K27M tumors had decreased H3K27me3 and H2AK119Ub, the repressive histone modification catalyzed by Polycomb Repressive Complex 1 (Figure 5C). To uncover genes deregulated by H3.3K27M, we performed RNA-seq of H3.3 and H3.3K27M melanomas. The majority of differentially expressed genes were upregulated in H3.3K27M tumors, including known PRC2 target genes of the *hox* and *fox* families (Figure 5D). Genes deregulated by *Eed* loss were similarly altered in H3.3K27M melanomas (Figure 5E). Expression of *ezh2* was decreased in H3.3K27M tumors (Figure 5F **,** *p* = 6.37E−06, log_2_ fold change [L2FC] = −1.52).

We hypothesized that high expression of genes upregulated by H3.3K27M in zebrafish tumors would be consistent with poor patient survival in humans. Toward this end, we identified a small set of genes (*n* = 50) that were significantly upregulated in H3.3K27M melanomas (Supplementary Figure S2A) and used these genes as a signature to interrogate a cohort of metastatic melanoma patients with clinical outcome data. We found that the gene signature identified in zebrafish stratifies overall and disease-free survival in human patients, with high expression of genes upregulated in H3.3K27M tumors associated with decreased survival (Figure 5G). Using ChIP-seq and transcriptional data from NIH Epigenomics Roadmap Data (Kundaje et al., 2015), we determined that 33/50 (66%) of genes in the signature are enriched with H3K27me3 and 36/50 (72%) are transcriptionally silenced in human melanocytes (Supplementary Figure S2B). These findings demonstrate that the genes deregulated by H3.3K27M in zebrafish are silenced PRC2 targets in humans and that aberrant upregulation of these targets is associated with poor patient prognosis.

### *FOXD1* is a PRC2 Target in Melanocytes

**FIGURE 6:**
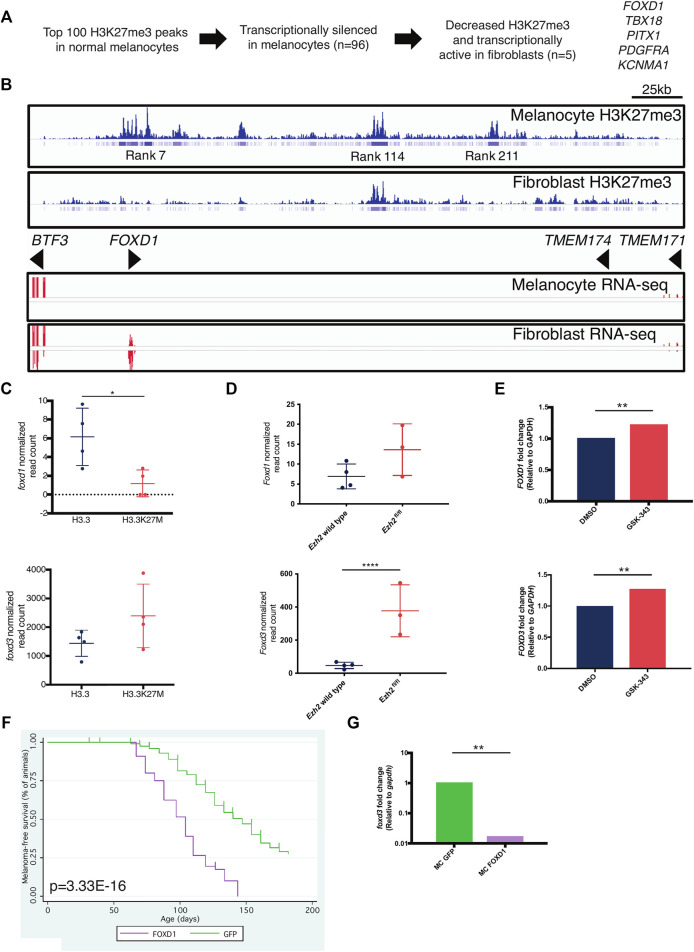
FOXD1 is a PRC2 target gene in melanocytes. **(A)** Scheme to identify melanocyte-specific PRC2 targets. **(B)** H3K27me3 (top) and RNA-seq (bottom) around the FOXD1 locus in melanocytes and fibroblasts. Rank indicates the rank of enrichment within the entire melanocyte genome ie, rank 7 is the seventh highest level of H3K27me3 enrichment in the melanocyte genome. **(C)** RNA-seq data of *foxd1* (top) and *foxd3* (bottom) levels within miniCoopR H3.3 and H3.3K27M zebrafish tumors. *foxd1*, *p* = 0.0397, L2FC = −1.33. *foxd3, p* = 0.101, L2FC = 0.656. **(D)** RNA-seq data of *Foxd1* (top) and *Foxd3* (bottom) levels in *Ezh2* wild-type and *Ezh2* ^fl/fl^ mouse tumors. *Foxd1*, *p* = 0.185, L2FC = 0.853. *Foxd3, p* = 4.49E-11, L2FC = 2.81. **(E)** qPCR data of *FOXD1* (top) and *FOXD3* (bottom) levels in A375 human melanoma cells treated with EZH2 inhibitor GSK-343. *FOXD1, p* = 0.0098, L2FC = 1.218. *FOXD3, p* = 0.0037, L2FC = 1.275. **(F)** Kaplan-Meier survival curves of Tg(mitfa:BRAF(V600E)); tp53^zdf1/zdf1^; mitfa^w2/w2^ zebrafish injected with indicated miniCoopR constructs. *p* value shown calculated from log-rank test between miniCoopR GFP and FOXD1. **(G)** qPCR data of *foxd3* levels in miniCoopR (MC) GFP and FOXD1 tumors, *p* = 0.0011.

To uncover PRC2 targets in melanocytes, we performed an in silico screen using Roadmap (Kundaje et al., 2015) data to identify genes with differential H3K27me3 and expression between melanocytes and fibroblasts. We sought to identify genes with *1*) high H3K27me3 and no expression in melanocytes and *2*) low H3K27me3 and expression in fibroblasts. From the top 100 H3K27me3 peaks in melanocytes, we identified five genes with differential H3K27me3 and expression (Figure 6A). *FOXD1*, our top hit, is extensively marked by H3K27me3 in melanocytes but not fibroblasts (Figure 6B). These data suggest that PRC2 is important in repressing expression of *FOXD1* in melanocytes.

FOXD1 and FOXD3 are members of the forkhead transcription factor family. FOXD1 is required for proper development of the kidneys and retina (Hatini et al., 1996; Herrera et al., 2004), and FOXD3 is required for neural crest specification (Stewart et al., 2006; Lister et al., 2006). Given the cell-type specific expression of FOXD genes, we hypothesized that PRC2 might play a role in regulating their expression across lineages. We examined the effects of decreased PRC2 activity on FOXD1/3 expression across several species. Unexpectedly, *foxd1* expression was decreased (*p* = 0.0397, L2FC = −1.33 and *foxd3* expression was modestly increased (*p* = 0.101, L2FC = 0.656) in miniCoopR H3.3K27M tumors (Figure 6C). We bred an *Ezh2* ^fl/fl^ allele (Su et al., 2005) onto the *Tyr::CreERT2*; *Braf* ^CA/wt^; *Pten* ^fl/fl^ melanoma strain (Dankort et al., 2009) and treated mice with 4-hydroxytamoxifen to induce recombination of alleles. Relative to tumors *Ezh2* wild-type tumors, tumors with *Ezh2* loss had increased *Foxd3* expression (*p* = 4.49E−11, L2FC = 2.81) and no significant change in *Foxd1* expression (*p* = 0.185, L2FC = 0.853) (Figure 6D). As expected, *FOXD1* read counts were nearly zero in all across both species tumors. We also treated A375 human melanoma cells with the EZH2 inhibitor GSK-343 (Verma et al., 2012) and verified decreased H3K27me3 (Supplementary Figure S3A). Inhibition of EZH2 increased levels of both *FOXD1* (*p* = 0.0098) and *FOXD3* (*p* = 0.0037) (Figure 6E). These data indicate that PRC2 regulates FOXD1/3 in the melanocyte lineage across multiple species.

### Overexpression of* FOXD1 *Accelerates Melanoma Onset and Decreases *FOXD3* Expression

Because *FOXD1* is not expressed in melanocytes (Figure 6B) and is minimally expressed in melanoma (Figures 6C,D), we hypothesized that overexpression of *FOXD1* would alter melanoma formation. To test this hypothesis, we used miniCoopR to overexpress *FOXD1* in melanoma. We found that ectopic expression of *FOXD1* accelerates melanoma onset (Figure 6F **,** *p* = 3.33E−16, log-rank). miniCoopR FOXD1 tumors had decreased expression of *foxd3* (*p* = 0.0011) (Figure 6G). These data indicate that aberrant *FOXD1* expression promotes tumorigenesis in melanocytes and leads to altered expression of *foxd3*.

## Discussion

Here we describe our work investigating the role of H3 HMTs in melanoma formation. We used oncogenic histone K-to-M mutations to model loss of function of H3K9 and H3K27 HMTs in a zebrafish melanoma model and further characterized the effects of H3 HMT loss using human and mouse systems. This study highlights the importance of integration of zebrafish systems into the comprehensive characterization of oncogenic alterations.

We have previously demonstrated that overexpression of H3K9 HMTs accelerated melanoma onset (Ceol et al., 2011), and in this study we investigate the effects of inhibition of H3K9 HMTs using H3.3K9M and A-366. Our findings further elucidate the connection between the H3K9 methyltransferases and the innate immune system. Epigenetic inhibitors have been described to induce transposable elements, thus activating the innate immune system and anti-tumor effects (Jones et al., 2019). Suppression of SETDB1 leads to the release of transposable elements and a type I interferon response in multiple systems (Cuellar et al., 2017; Kato et al., 2018; Zhang et al., 2021). In addition, SETDB1 loss has been demonstrated to activate cytotoxic T cell responses *in vivo* (Griffin et al., 2021). In our study, transposable elements were upregulated in H3.3K9M tumors, thus highlighting the role of H3K9 HMTs in maintaining homeostasis of these elements. In another study, inhibition of G9a in fibroblasts led to type I interferon production and expression of interferon stimulated genes (Fang et al., 2012). In our studies we found that inhibition of H3K9 HMTs through either H3.3K9M or A-366 activated innate immune system signatures. Further, decreased H3K9 methylation increased chromatin accessibility at CD33, a gene involved in the neutrophil degranulation pathway, and resulted in increased expression of *CD33*. Thus we demonstrate that inhibition of H3K9 HMTs leads to an axis of release of transposable elements as well as activation of the innate immune system in a tumor setting.

We observed a neutrophil response in a cell-independent manner in sorted H3.3K9M-mScarlet cells, suggesting that melanoma cells activate an immune response that can include neutrophil chemotaxis when H3K9M HMTs are inhibited by H3.3K9M or A-366. We also found that the CD33 locus was more accessible and expression was increased in H3.3K9M-mScarlet cells. Although there is no CD33 orthologue in zebrafish, we observed enrichment in neutrophil pathways in H3.3K9M tumors, further suggesting a link between H3K9 HMTs and neutrophil response. Future studies inhibiting neutrophil chemotaxis could provide a more direct understanding of the relationship between H3K9 HMTs and the innate immune system.

In contrast to H3K9 HMTs, components of PRC2 are subject to both gain and loss of function alterations in human melanoma. In our system, H3.3K27M accelerated melanoma onset, but activating EZH2 mutations have been shown to promote melanoma onset in mice (Souroullas et al., 2016). These differences may be the result of experimental differences, such as the use of a knock-in alleles in the mouse model versus our overexpression model. More broadly, in light of these data and human genetic data, these results suggest that PRC2 function in melanoma may be context-specific, so PRC2 therapeutic strategies in melanoma may not be uniform. High expression of genes deregulated in H3.3K27M melanomas was associated with poor prognosis in human melanoma, indicating that the programs regulated by PRC2 are conserved across species.

We also uncovered a FOXD regulatory network regulated by PRC2 in zebrafish, mice, and humans. We identified *FOXD1* as a PRC2 target that is marked by H3K27me3, silenced in melanocytes, and expressed minimally in zebrafish and mouse melanomas. Overexpression of *FOXD1* using miniCoopR accelerated melanoma onset—the first demonstration that FOXD1 is oncogenic in any lineage—thereby highlighting the importance of *FOXD1* silencing in melanoma. We also observed changes in FOXD1/3 expression in zebrafish, mouse, and human systems engineered with reduction in PRC2 activity, indicating that the mechanisms regulating FOXD1/3 expression are conserved across species. Although *FOXD1* is extensively marked by H3K27me3 in melanocytes, we did not observe significant increases in *FOXD1* expression in two *in vivo* melanoma models with PRC2 loss of function, possibly because the epigenetic programs that repress *FOXD1* expression extend beyond H3K27me3. *foxd1* expression was increased in miniCoopR H3.3K27M tumors, suggesting that regulation of expression may be multifaceted. Future chromatin immunoprecipitation (ChIP) experiments may better illuminate the precise dynamics between PRC2, *FOXD1*, and *FOXD3*.

Our work broadly demonstrates the utility of using H3 K-to-M alleles to interrogate H3 HMT function *in vitro* and *in vivo*. Expression of K-to-M alleles induced changes in chromatin structure and gene expression across systems and produced tumor phenotypes in zebrafish that are genetically consistent with human disease. Given that multiple HMTs are known to catalyze methylation at each locus, K-to-M mutations are an ideal tool to probe the role of H3 methylation at specific sites. Future work using CRISPR/Cas9 to evaluate loss of H3 HMTs alone and in combination will provide additional insight into the role of specific HMTs in disease and developmental states. Because there are likely to be other effects of H3 K-to-M mutations, CRISPR/Cas9 loss of function studies will also allow for the assessment of the direct effects of HMT loss of function. These studies will be critical in the development of personalized epigenetic therapy in melanoma and other neoplasms.

## Data Availability

The data presented in the study are deposited in the Gene Expression Omnibus (GEO) repository, accession numbers GSE192439, GSE192476, GSE192612, GSE192436, GSE192491 and GSE194422.
