# Supplementary figures and images for: Histone 3 Methyltransferases Alter Melanoma Initiation and Progression Through Discrete Mechanisms
Source: Front Cell Dev Biol. 2022 Feb 10;10:814216. doi: 10.3389/fcell.2022.814216 (PMC8866878; doi:10.3389/fcell.2022.814216)

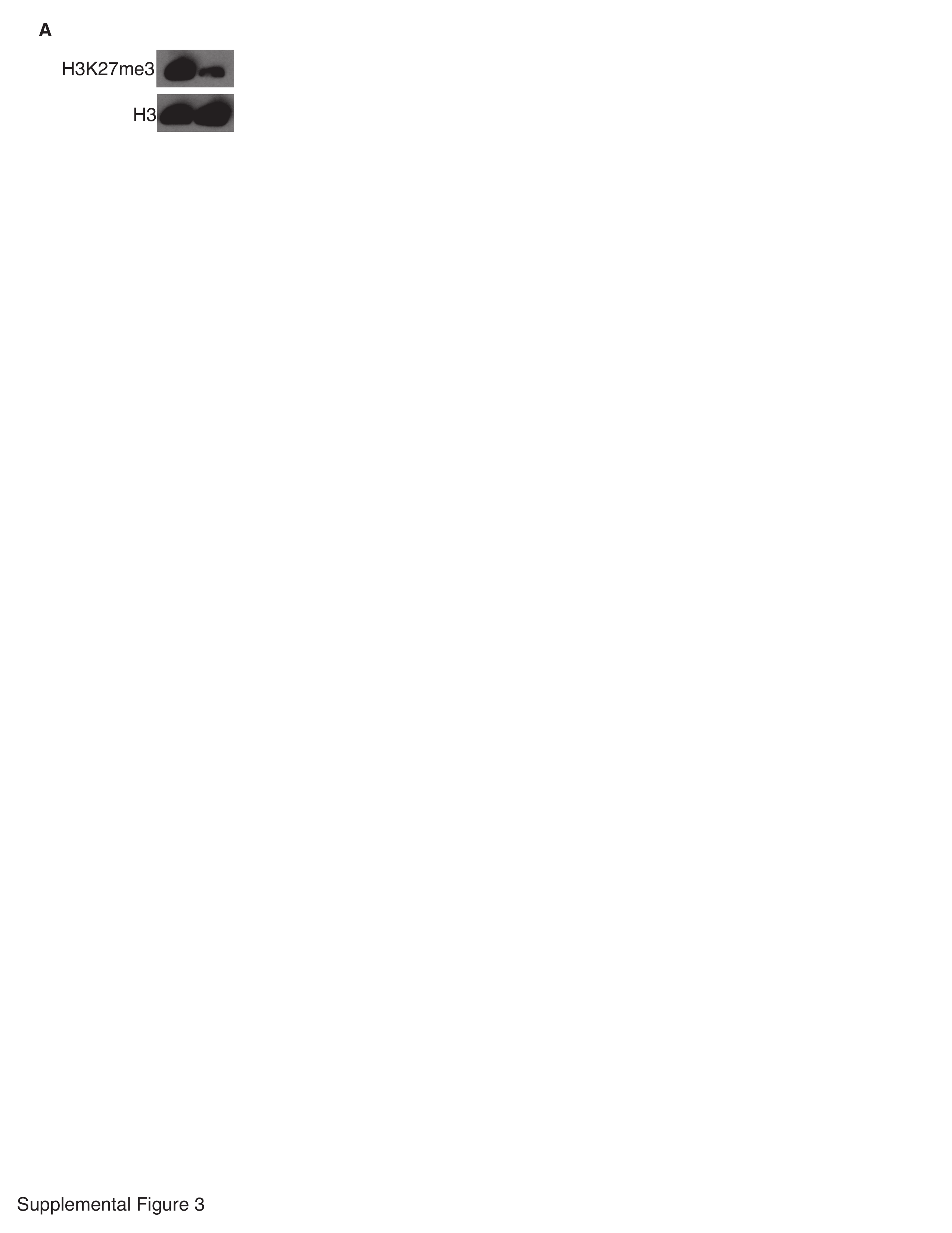

Supplement: Supplementary file 1 [file Image3.TIFF]

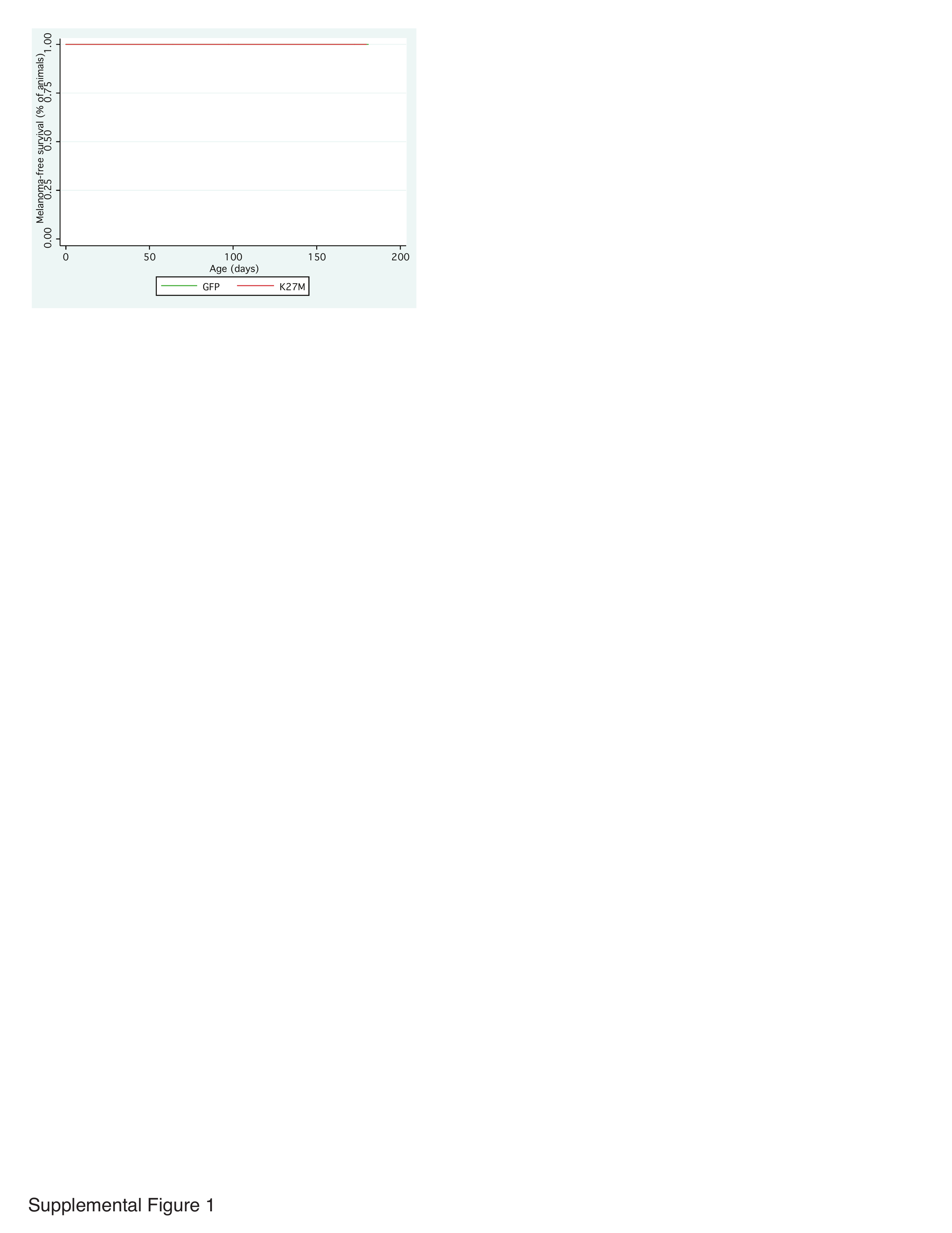

Supplement: Supplementary file 2 [file Image1.TIFF]

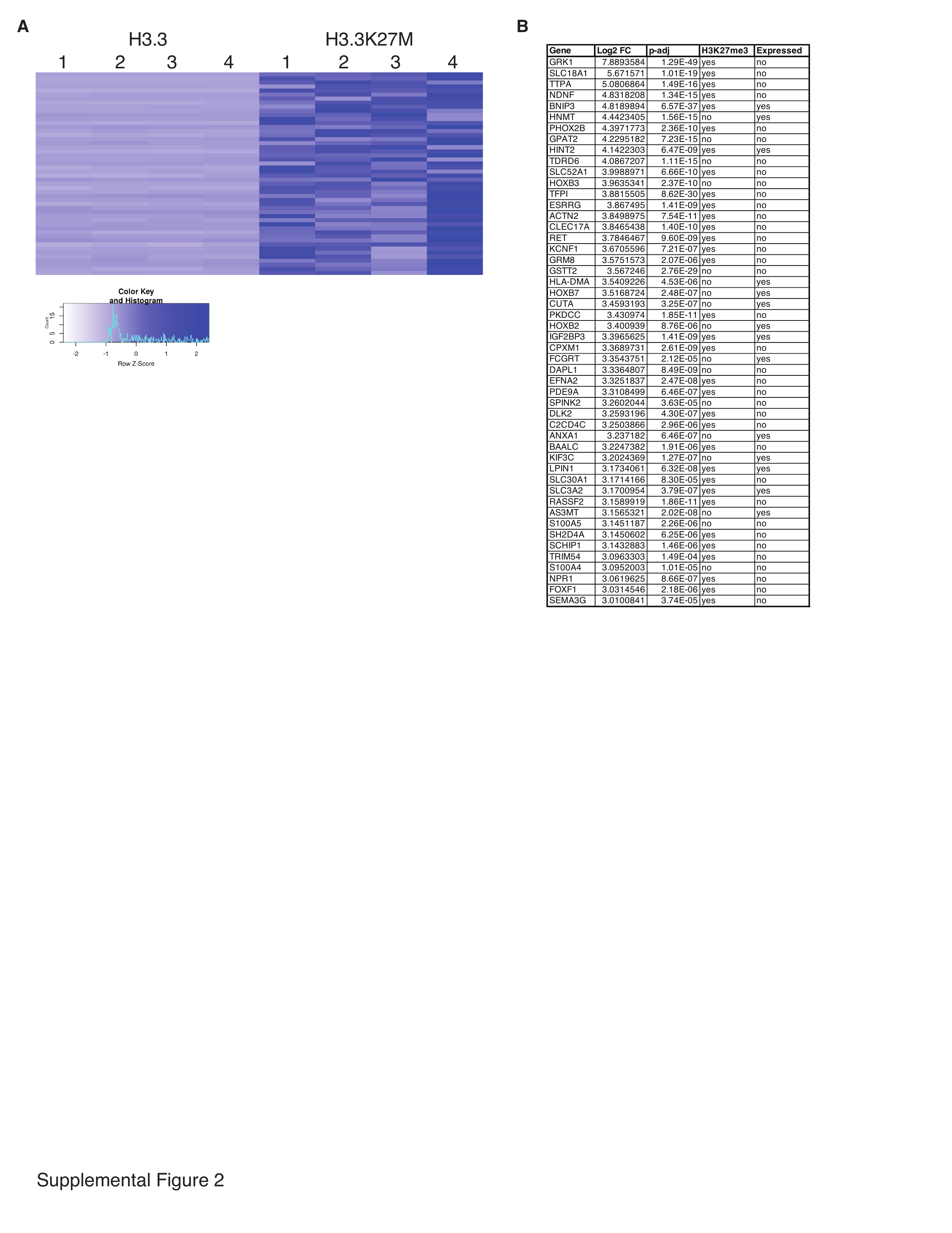

Supplement: Supplementary file 3 [file Image2.TIFF]
